# Longitudinal Changes in Peripapillary Retinal Nerve Fiber Layer and Macular Ganglion Cell Inner Plexiform Layer in Progressive Myopia and Glaucoma Among Adolescents

**DOI:** 10.3389/fmed.2022.828991

**Published:** 2022-03-22

**Authors:** Hui Xiao, Yimin Zhong, Yunlan Ling, Xiaoyu Xu, Xing Liu

**Affiliations:** State Key Laboratory of Ophthalmology, Zhongshan Ophthalmic Center, Sun Yat-sen University, Guangdong Provincial Key Laboratory of Ophthalmology and Visual Science, Guangdong Provincial Clinical Research Center for Ocular Diseases, Guangzhou, China

**Keywords:** adolescent, glaucoma, myopia, retinal nerve fiber layer, ganglion cell plus inner plexiform layer

## Abstract

**Purpose:**

This study aimed to investigate the differences in longitudinal changes in the peripapillary retinal nerve fiber layer (pRNFL) and macular ganglion cell plus inner plexiform layer (GCIPL) caused by progressive myopia and glaucoma among adolescents.

**Design:**

This was a retrospective observational study.

**Methods:**

A total of forty-seven and 25 eyes of 47 and 25 adolescents with myopia progression (MP) and glaucoma progression (GP), respectively, who were followed up at the Zhongshan Ophthalmic Center for at least 3 years, were included in the study. The pRNFL and GCIPL that measured at the initial and last visits were analyzed.

**Results:**

The median follow-up period was 5 years for both two groups. During follow-up, the whole, superior, and inferior pRNFL decreased in both the MP and GP groups, (*p* < 0.001). Nasal pRNFL decreased in the MP group (*p* < 0.001) but had no significant difference in the GP group (*p* = 0.19). Temporal pRNFL was increased in the MP group (*p* < 0.001) but decreased in the GP group (*p* < 0.001). The average and sectoral GCIPL decreased in both groups (*p* < 0.001). The annual change rate of temporal pRNFL and pRNFL at 10-, 8-, 9-, and 7-clock-hour sectors and the inferotemporal GCIPL has better diagnostic value to differentiate glaucoma from myopia (the area under the receiver operating characteristic curve, AUC > 0.85).

**Conclusion:**

Glaucoma and MP could cause loss of the pRNFL and GCIPL in adolescents; however, the loss patterns were different between the two groups. The temporal quadrant and 7-, 8-, 9-, and 10-clock-hour sector pRNFL and the inferotemporal GCIPL can help distinguish pRNFL and GCIPL loss caused by glaucoma or MP.

## Introduction

Glaucoma is the leading cause of irreversible blindness (1) and is characterized by the degeneration of the retinal ganglion cells (RGCs), with progressive loss of the retinal nerve fiber layer (RNFL) and the corresponding visual field (2). The previous studies (3, 4) have shown that the loss of the peripapillary retinal nerve fiber layer (pRNFL) and RGCs was more sensitive than the visual field test in detecting early glaucoma and the progression of glaucoma and was therefore a sensitive indicator for detecting glaucoma in the general population. However, the studies (5–7) have shown that myopia could also lead to the loss of pRNFL and RGCs, which makes it difficult to determine whether the loss of pRNFL is due to myopia or early glaucoma (8). As glaucoma is a chronic progressive neuropathy, longitudinal observation of the loss of pRNFL and RGCs in suspected cases is necessary to differentiate glaucoma from myopia. However, when it comes to adolescents, the problem becomes more confused. As myopia typically manifests and progresses in childhood and adolescence (9), the possible impact of myopia development and its effect on the RNFL and RGCs in adolescent myopia cannot be ignored. Previous cross-sectional studies (10, 11) reported a significant correlation between pRNFL and the spherical equivalent (SE) in Chinese and Caucasian students. One study (12) using long-wavelength swept-source optical coherence tomography (OCT) also found that the macular inner plexiform layer (IPL) plus ganglion cell layer (GCL) in children with myopia exhibited a small but statistically significant decrease over the course of 18 months. Thus, identifying the difference in the loss pattern of the pRNFL and RGCs caused by glaucomatous or myopic progression in adolescents is critical for the early detection of juvenile glaucoma. To date, most studies (6, 13) that have compared the pRNFL and RGCs between myopia and glaucoma were cross-sectional investigations on adults, which limits the insights that can be drawn from the data regarding the time course of pRNFL and RGCs changes associated with the progressive myopia and glaucoma during adolescence.

Therefore, this study retrospectively analyzed the long-term follow-up data of pRNFL and the ganglion cell plus inner plexiform layer (GCIPL) in patients with adolescent myopia and juvenile glaucoma at the Zhongshan Ophthalmic Center from September 2013 to December 2020. We compared the difference in loss patterns of the pRNFL and macular GCIPL between progressive myopia and glaucoma among adolescents to provide useful information that could differentiate glaucomatous progression from myopic progression.

## Materials and Methods

### Study Participants

This retrospective observational study was approved by the Ethics Review Committee of the Zhongshan Ophthalmic Center and adhered to the tenets of the Declaration of Helsinki for research involving human subjects. Informed consent was obtained from the guardians of all participants involved in the study.

We retrospectively reviewed the medical records of patients with myopia and glaucoma in adolescents at the Zhongshan Ophthalmic Center, Sun Yat-sen University, between September 2013 and December 2020. The enrolled participants have been followed up for at least 3 years, with the complete routine ocular examination records and adequate quality pRNFL and macular GCIPL measurements at the initial and last visits.

The routine ocular examination records included comprehensive ophthalmological examination, cycloplegic refraction, best-corrected visual acuity (BCVA), intraocular pressure (IOP) measurement using a Goldmann applanation tonometer, and disk photography (Kowa non-myd a-D III; Kowa Optimed Inc., Aichi, Japan). The RNFL and GCIPL were obtained using OCT.

The inclusion criteria for progressive myopia in adolescents were as follows: (1) age <18 years at initial visit; (2) progression of myopia (SE) more than 1 D during the follow-up; (3) BCVA ≥20/25, with IOP <21 mmHg at each visit; (4) no glaucomatous optic neuropathy (GON) confirmed and agreed upon by two glaucoma specialists based on disk photography during the follow-up; and (5) no family history of glaucoma or intraocular surgery.

The inclusion criteria for progressed glaucoma in adolescents were as follows: (1) age <18 years at initial visit; (2) IOP >22 mmHg for at least two times measurements by Goldmann applanation tonometer, without treatment; (3) open angle; and (4) glaucoma progression (GP) confirmed by progression of the optic nerve defects during the follow-up. The GON progression was determined through the evaluation of the entire series of stereoscopic optic disk and red-free RNFL photographs by two glaucoma specialists (HX and XL) independently. The most recent photograph of each patient was compared with the baseline photograph. GP was defined as an increase in the extent of neuroretinal rim thinning, enhancement of disk excavation, and/or any widening, deepening, or new RNFL defects. The graders classified each glaucomatous eye as either stable or progressive. Cases were excluded if the opinions of the two observers differed.

The exclusion criteria were as follows: (1) blurred disk photography that cannot be evaluated; (2) poor OCT quality, defined by signal strength <6 or poor alignment on the individual OCT scans; (3) other retinal, uveal, neurological, or systemic diseases that could affect retinal results; and (4) prior ocular trauma and intraocular surgery history, except for glaucoma surgery. If data from both eyes were eligible for analysis, only one randomly selected eye from each patient was included in this study.

### Peripapillary Retinal Nerve Fiber Layer and Macular Ganglion Cell Plus Inner Plexiform Layer Measurements

Measurements of the pRNFL and GCIPL were taken during the follow-up using Cirrus HD-OCT 5000 (Carl Zeiss Meditec, Inc., Dublin, CA, United States; version 10.0) and were subsequently analyzed. An optic disk cube 200 × 200 protocol centered at the optic disk center and a macular cube 512 × 128 scan protocol centered at the fovea were performed for all eyes. Participants were tested using the same device during the follow-up examinations.

The cup/disk area ratio (C/D) was automatically generated by the optic nerve head (ONH) algorithm used in Cirrus OCT software from the optic disk cube 200 × 200 protocol. The pRNFL software automatically extracted a peripapillary circle (3.46 mm in diameter) from the cube data set to measure pRNFL thickness. The average, four quadrants, and 12-clock-hour RNFL thickness were analyzed in this study. All of the clock positions for the left eye were converted to the right eye clock position and were recorded (i.e., the pRNFL of 1 o’clock for the left eye was recorded as 11 o’clock and 11 o’clock for the left was recorded as 1 o’clock).

The ganglion cell analysis algorithm was used to process the data obtained with the macular cube 512 × 128 scan protocol to calculate the macular GCIPL thickness within a 14.13-mm^2^ elliptical annular area centered at the fovea. The algorithm identified the distance between the outer boundary of the RNFL and the IPL as the thickness of the GCIPL. The average and six sectoral (superior, superonasal, inferonasal, inferior, inferotemporal, and supertemporal) GCIPL thickness measurements were analyzed.

### Statistical Analysis

Statistical analyses were performed using SPSS (version 20.0; SPSS Inc., Chicago, IL, United States). The Kolmogorov–Smirnov test was performed to test for normality. An independent *t*-test or Mann–Whitney *U* test (for non-parametric data) was used to compare the baseline data between the myopia and glaucoma groups. Baseline and follow-up pRNFL and GCIPL data were compared in each group using the pairwise *t*-test. The annual change rate was calculated as [(last visit–initial visit)/follow-up duration (years)]. General linear regression model was used to describe the association between the group and the annual change rate of the whole and regional RNFL/GCIPL, and the coeffect of initial age, initial refractive error, and annual refractive error change rate were also considered in the model. The area under the receiver operating characteristic curve (AUC) was used to evaluate the diagnostic value of the annual change rate of the mean and sectoral pRNFL and GCIPL between myopia and glaucoma groups. The significance level was set to 0.05 for a two-sided alternative hypothesis test.

## Results

A total of forty-seven eyes of 47 subjects in the adolescent myopia progression (MP) group and 25 eyes of 25 subjects in the GP group were included. The basic demographics of the two groups are listed in Table 1.

**TABLE 1:** Basic demographics of progressive myopia and progressive glaucoma eyes in adolescents.

	Myopia	Glaucoma	*P*
Number	47	25	−
Sex (Female, %)	41.55%	40.00%	−
Laterality (Right, %)	57.45%	60.00%	−
Age at initial visit (years, mean ± SD)	10.97 ± 2.30	12.76 ± 3.92	0.05*
SE at initial visit (D, mean ± SD)	−1.89 ± 0.92	−2.48 ± 1.74	0.12*
BCVA at initial visit (logMAR, mean ± SD)	−0.05 ± 0.07	−0.03 ± 0.07	0.64*
IOP at initial visit (mmHg, mean ± SD)	15.93 ± 1.83	23.77 ± 2.87	<0.001*
Follow-up period (years, median, IQR)	5 (4∼6)	5 (3∼6)	0.80^†^
Age at last visit (years, mean ± SD)	14.92 ± 2.70	17.00 ± 3.74	0.02*
SE at last visit (D, mean ± SD)	−4.60 ± 1.61	−4.64 ± 2.80	0.95*
BCVA at last visit (logMAR, mean ± SD)	−0.05 ± 0.07	0.01 ± 0.07	0.24*
IOP at last visit (mmHg, mean ± SD)	15.89 ± 1.78	18.38 ± 3.85	0.11*

*logMAR, logarithm of the minimum angle of resolution; SE, spherical equivalent; BCVA, best-corrected visual acuity; IOP, intraocular pressure; SD, standard deviation, IQR, interquartile range.*

*Independent t-test.

*^†^Mann–Whitney U test.*

**FIGURE 1:**
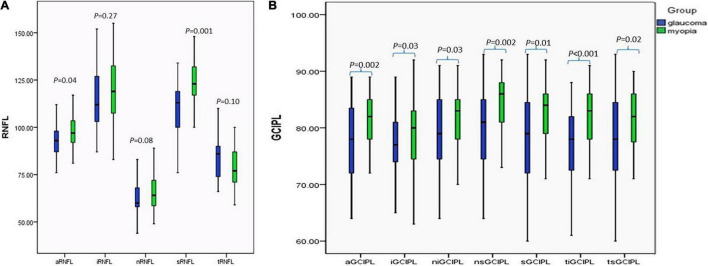
Comparison of the pRNFL and the macular GCIPL between the glaucoma and myopia groups at initial visit. **(A)** Comparison of the pRNFL between the glaucoma and myopia groups at initial visit. **(B)** Comparison of the macular GCIPL between the glaucoma and myopia groups at initial visit. aRNFL, average retinal nerve fiber layer; iRNFL, inferior retinal nerve fiber layer; nRNFL, nasal retinal nerve fiber layer; sRNFL, superior retinal nerve fiber layer; tRNFL, temporal retinal nerve fiber layer; aGCIPL, average ganglion cell plus inner plexiform layer complex; iGCIPL, inferior ganglion cell and inner plexiform layer complex; niGCIPL: inferonasal ganglion cell plus inner plexiform layer complex; nsGCIPL, superonasal ganglion cell plus inner plexiform layer complex; sGCIPL, superior ganglion cell plus inner plexiform layer complex; tiGCIPL, inferotemporal ganglion cell plus inner plexiform layer complex; tsGCIPL, supertemporal ganglion cell plus inner plexiform layer complex.

At the initial visit, the whole average and superior pRNFL were thinner in the glaucoma group (*p* < 0.05), whereas no statistically significant difference was found in the inferior, temporal, and nasal pRNFL between the two groups (Figure 1A), whereas the average and sectoral GCIPL in the glaucoma group were thinner than myopia group (*p* < 0.05) (Figure 1B).

**FIGURE 2:**
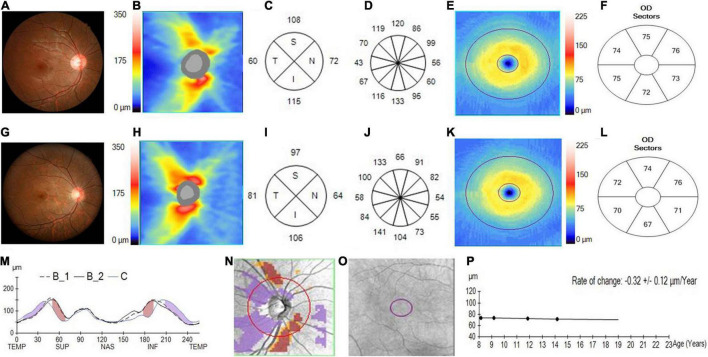
Changes in the pRNFL and macular GCIPL in an adolescent with progressed myopia. **(A)** The fundus photograph of an adolescent myopia taken at initial visit in 2013. **(B)** The pRNFL thickness map taken at the initial visit. **(C)** The quadrantal pRNFL thickness at the initial visit. **(D)** The clock-hour pRNFL thickness at the initial visit. **(E)** The GCIPL thickness map taken at the initial visit. **(F)** The sectoral GCIPL thickness at the initial visit. **(G)** The fundus photograph of adolescent myopia taken at the last visit in 2018. **(H)** The pRNFL thickness map taken at the last visit. **(I)** The quadrantal pRNFL thickness at the last visit. **(J)** The clock-hour pRNFL thickness at the last visit. **(K)** The GCIPL thickness map taken at the last visit. **(L)** The sectoral GCIPL thickness at the last visit. **(M)** The pRNFL thickness profiles showing that the pRNFL thickness was possibly increased in the temporal quadrant (purple region), but was likely decreased in the superior and inferior quadrants (brown region) during the follow-up (B1, baseline 1; B2, baseline 2; C, current line). **(N)** The change possibility map of pRNFL thickness showing that the pRNFL thickness was possibly increased in the temporal quadrant (purple region), but was likely decreased in the superior and inferior quadrants (brown region) during the follow-up. **(O)** The change possibility map of GCIPL thickness showing neither possible increase nor possible loss in the entire GCIPL measured region during the follow-up. **(P)** The change trend line of GCIPL thickness profiles showing that the change rate of GCIPL was about –0.32 ± 0.12 μm/year.

**FIGURE 3:**
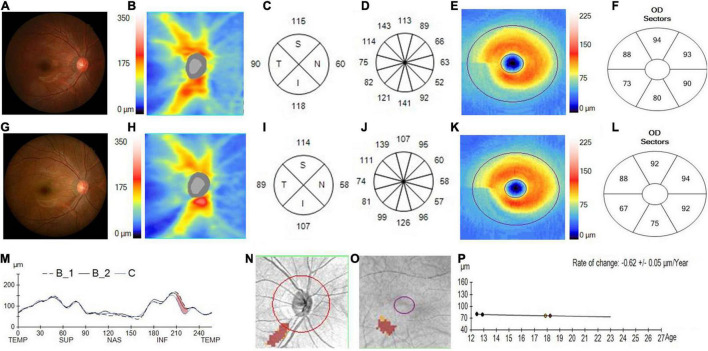
Changes in the pRNFL and macular GCIPL in a progressed juvenile glaucoma. **(A)** The fundus photograph of a juvenile glaucoma case taken at the initial visit in 2014, with the white arrow showing a wedge defect of the RNFL corresponding to the narrowed inferior rim. **(B)** The pRNFL thickness map taken at the initial visit. **(C)** The quadrantal RNFL thickness at the initial visit. **(D)** The clock-hour RNFL thickness at the initial visit. **(E)** The GCIPL thickness map taken at the initial visit. **(F)** The sectoral GCIPL at the initial visit. **(G)** The fundus photograph of a juvenile glaucoma case taken at the last visit in 2019, with a considerable serious defect of the RNFL and narrower rim displayed on the photograph. **(H)** The RNFL thickness map taken at the last visit. **(I)** The quadrantal RNFL thickness at the last visit. **(J)** The clock-hour RNFL thickness at the last visit. **(K)** The GCIPL thickness map taken at the last visit. **(L)** The sectoral GCIPL at the last visit. **(M)** The pRNFL thickness profiles showing that the RNFL thickness was likely decreased in the inferotemporal region (brown region) during the follow-up (B1, baseline 1; B2, baseline 2; C, current line). **(N)** The change possibility map of RNFL thickness showing that the RNFL thickness was likely decreased in the inferotemporal region (brown region) during the follow-up. **(O)** The change possibility map of GCIPL thickness showing that GCIPL was likely decreased in the inferotemporal region (brown region) during the follow-up. **(P)** The change trend line of GCIPL thickness profiles showing that the change rate of GCIPL was about –0.62 ± 0.05 μm/year.

The pRNFL and GCIPL of the initial and last visits in the myopia and GP groups are shown in Figures 2, 3 and Table 2. In both the myopia and glaucoma groups, the average thickness of the whole, superior, and inferior pRNFL decreased during follow-up (*p* < 0.05). Nasal pRNFL decreased in the myopia group (*p* < 0.05) during the follow-up period, but no statistically significant difference was observed in the glaucoma group (*p* = 0.19) between the initial and last visits. The temporal pRNFL was increased in the MP group (*p* < 0.05), but decreased in the GP group during follow-up (*p* < 0.05). In the MP group, the pRNFL thickness at the 10, 9, 8, and 7 o’clock sections increased during the follow-up, whereas the pRNFL thickness at the 12, 1, 2, 5, and 6 o’clock sections decreased. By contrast, no statistically significant differences were found in the pRNFL thickness at the 3, 4, and 11 o’clock sections during follow-up (*p* = 0.08, 0.08, and 0.57). In the GP group, the pRNFL thickness decreased at most of the clock-hour sections during follow-up, except at the 4 and 3 o’clock sections. The average and sectoral GCIPL became thinner during follow-up in both myopia and glaucoma groups (*p* < 0.05).

**TABLE 2:** Comparison of the pRNFL and GCIPL between the initial and last visits.

	Myopia (mean ± SD) μ m			Glaucoma (mean ± SD) μ m		
		
	Initial visit	Last visit	*P*	Initial visit	Last visit	*P*
*N* (eye)	47	47		25	25	
Average RNFL	97.53 ± 8.60	94.78 ± 8.70	<0.001*	93.00 ± 9.12	81.12 ± 12.28	<0.001*
Superior RNFL	123.81 ± 12.26	115.31 ± 12.62	<0.001*	110.20 ± 14.50	95.24 ± 19.19	<0.001*
Temporal RNFL	79.38 ± 12.74	92.80 ± 14.59	<0.001*	85.20 ± 14.58	74.75 ± 10.19	0.01*
Inferior RNFL	119.64 ± 16.72	112.67 ± 14.73	<0.001*	115.00 ± 17.51	93.52 ± 20.21	<0.001*
Nasal RNFL	65.64 ± 8.99	61.34 ± 8.78	<0.001*	61.56 ± 10.49	60.68 ± 11.70	0.19
RNFL12′	121.21 ± 20.43	109.75 ± 20.84	<0.001*	107.80 ± 18.23	93.16 ± 22.45	<0.001*
RNFL11′	140.06 ± 20.02	141.58 ± 17.88	0.57	128.96 ± 24.69	111.00 ± 29.77	<0.001*
RNFL10′	92.28 ± 16.71	106.96 ± 20.71	<0.001*	97.24 ± 27.95	83.56 ± 18.94	0.01*
RNFL9′	61.54 ± 10.20	70.13 ± 10.19	<0.001*	64.44 ± 13.83	60.20 ± 12.66	0.10
RNFL8′	84.40 ± 17.75	100.31 ± 18.80	<0.001*	91.24 ± 12.16	80.36 ± 14.39	0.001*
RNFL7′	149.22 ± 23.61	154.55 ± 19.03	0.01*	144.48 ± 12.50	111.88 ± 24.68	<0.001*
RNFL6′	121.38 ± 28.07	106.78 ± 25.40	<0.001*	112.12 ± 25.83	92.88 ± 30.76	<0.001*
RNFL5′	88.82 ± 17.13	78.78 ± 14.30	<0.001*	85.20 ± 24.42	77.04 ± 25.42	0.03*
RNFL4′	57.74 ± 11.11	56.65 ± 11.11	0.08	55.80 ± 10.90	55.72 ± 10.46	0.98
RNFL3′	60.32 ± 10.94	57.62 ± 11.71	0.08	56.76 ± 10.40	57.52 ± 10.98	0.74
RNFL2′	75.74 ± 11.59	70.16 ± 12.58	<0.001*	74.08 ± 15.98	69.04 ± 17.22	0.06
RNFL1′	109.70 ± 18.22	95.82 ± 17.87	<0.001*	95.24 ± 17.16	81.16 ± 18.48	<0.001*
Average GCIPL	81.75 ± 4.94	78.84 ± 4.65	<0.001*	77.96 ± 7.20	71.33 ± 8.35	<0.001*
Superior GCIPL	82.85 ± 5.24	80.13 ± 4.86	<0.001*	79.96 ± 9.35	73.08 ± 11.96	0.002*
Superotemporal GCIPL	81.36 ± 5.08	79.78 ± 4.71	0.002*	77.91 ± 7.46	71.95 ± 9.91	<0.001*
Inferotemporal GCIPL	81.53 ± 5.72	78.89 ± 4.98	<0.001*	77.13 ± 7.58	65.96 ± 6.92	<0.001*
Inferior GCIPL	78.84 ± 6.33	74.67 ± 5.94	<0.001*	77.08 ± 6.54	67.83 ± 7.76	<0.001*
Inferonasal GCIPL	81.64 ± 5.44	76.63 ± 11.06	<0.001*	79.13 ± 7.32	73.79 ± 10.49	0.001*
Superonasal GCIPL	84.53 ± 5.05	81.31 ± 4.83	<0.001*	79.57 ± 8.22	73.83 ± 10.96	<0.001*

*RNFL, retinal nerve fiber layer; GCIPL, ganglion cell plus inner plexiform layer.*

*p < 0.05 for a two-sided alternative hypothesis test.

When the coeffect of initial age, initial refractive error, and annual refractive error change rate were considered, the annual change rates of most pRNFL parameters still showed significantly different between the myopia and glaucoma groups, except the nasal pRNFL and pRNFL at 1–5 and 11 o’clock. In terms of GCIPL, the annual change rates of average, superotemporal, inferotemporal, and inferior GCIPL had significant difference between the two groups (Table 3).

**TABLE 3:** Annual change rate of RNFL and GCIPL between Glaucoma and Myopia groups.

Annual change rate	Myopia group (Mean ± SD) *N* = 47	Glaucoma group (Mean ± SD) *N* = 25	β coefficient (95% CI)	*p*
**RNFL (μ m/year)**				
Average RNFL	−0.55 ± 0.72	−3.55 ± 4.17	−3.30 (−4.61 ∼−2.00)	< 0.001*
Superior RNFL	−1.81 ± 1.95	−3.78 ± 5.01	−2.24 (−4.17 ∼−0.68)	0.007*
Temporal RNFL	3.33 ± 2.27	−2.99 ± 3.76	−6.10 (−7.55 ∼−4.65)	< 0.001*
Inferior RNFL	−1.73 ± 1.97	−6.50 ± 7.04	−5.39 (−7.72 ∼−3.05)	< 0.001*
Nasal RNFL	−0.81 ± 1.75	−0.51 ± 3.54	0.01 (−1.22 ∼ 1.23)	0.99
RNFL12′	−2.36 ± 3.64	−4.11 ± 4.59	−2.54 (−4.58∼−0.50)	0.02*
RNFL11′	0.59 ± 3.03	−2.46 ± 11.90	−3.02 (−0.6.50 ∼ 0.45)	0.09
RNFL10′	3.75 ± 3.14	−3.12 ± 4.17	−6.43 (−8.15∼−4.71)	< 0.001*
RNFL9′	2.16 ± 1.69	−1.16 ± 2.47	−3.13 (−4.11∼−2.15)	< 0.001*
RNFL8′	3.82 ± 3.30	−3.71 ± 4.89	−7.08 (−9.16 ∼−5.00)	< 0.001*
RNFL7′	1.07 ± 3.69	−10.15 ± 10.00	−10.74 (−14.25 ∼−7.23)	< 0.001*
RNFL6′	−3.65 ± 3.87	−5.94 ± 6.08	−3.27 (−5.68 ∼ 0.86)	0.01*
RNFL5′	−2.21 ± 2.61	−2.22 ± 5.31	−0.58 (−2.54 ∼ 1.39)	0.56
RNFL4′	−1.44 ± 2.07	−0.66 ± 2.45	0.42 (−0.74 ∼ 1.57)	0.47
RNFL3′	−1.62 ± 2.87	−0.38 ± 3.77	0.84 (−0.84 ∼ 2.52)	0.32
RNFL2′	−1.00 ± 2.39	−1.55 ± 4.16	−0.73 (−2.37 ∼ 0.92)	0.38
RNFL1′	−3.02 ± 3.37	−3.55 ± 3.69	−1.06 (−2.89 ∼ 0.77)	0.25
**GCIPL (μ m/year)**				
Average GCIPL	−0.69 ± 0.73	−1.68 ± 1.64	−1.07 (−1.67∼− 0.48)	0.001*
Superior GCIPL	−0.63 ± 1.05	−1.48 ± 2.60	−0.87 (−1.80 ∼ 0.06)	0.07
Superotemporal GCIPL	−0.37 ± 0.69	−1.36 ± 1.51	−0.87 (−1.42 ∼ 0.32)	0.002*
Inferotemporal GCIPL	−0.67 ± 0.66	−2.93 ± 2.89	−2.40 (−3.34 ∼−1.45)	< 0.001*
Inferior GCIPL	−0.98 ± 0.83	−2.40 ± 2.69	−1.70 (−2.60 ∼−0.81)	< 0.001*
Inferonasal GCIPL	−1.47 ± 4.05	−1.18 ± 1.90	−0.40 (−1.40 ∼ 2.21)	0.66
Superonasal GCIPL	−0.67 ± 0.82	−1.14 ± 1.21	−0.58 (−1.09 ∼−0.07)	0.05

*RNFL, retinal nerve fiber layer; GCIPL, ganglion cell plus inner plexiform layer.*

*p < 0.05 for a two-sided alternative hypothesis test.

The AUCs of the annual change rate of pRNFL and GCIPL are shown in Table 4. The AUCs of the annual change rate of pRNFL at the 10, 8, 9, and 7 o’clock sections, and also the temporal pRNFL and inferotemporal GCIPL, were above 0.85, which suggested that these parameters might be useful in differentiating whether the loss of pRNFL and GCIPL was caused by GP or MP (Table 4).

**TABLE 4:** The area under ROC curve (AUC) of the annual change rate of the pRNFL and the macular GCIPL to discriminate GP from MP.

Variable	Area under	Variable	Area under
	ROC curve		ROC curve
Average RNFL	0.79 (0.66∼0.92)	RNFL12′	0.58 (0.44∼0.73)
Superior RNFL	0.64 (0.48∼0.79)	RNFL11′	0.76 (0.64∼0.89)
Temporal RNFL	0.81 (0.72∼0.91)	RNFL10′	0.96 (0.92∼1.00)*
Inferior RNFL	0.99 (0.90∼1.00)*	RNFL9′	0.91 (0.83∼0.98)*
Nasal RNFL	0.45 (0.29∼0.60)	RNFL8′	0.94 (0.89∼0.99)*
Average GCIPL	0.66 (0.52∼0.80)	RNFL7′	0.95 (0.90∼0.99)*
Superior GCIPL	0.58 (0.43∼0.73)	RNFL6′	0.61 (0.48∼0.74)
Superotemporal GCIPL	0.69 (0.54∼0.83)	RNFL5′	0.45 (0.30∼0.61)
Inferotemporal GCIPL	0.89 (0.81∼0.96)*	RNFL4′	0.62 (0.49∼0.76)
Inferior GCIPL	0.64 (0.49∼0.78)	RNFL3′	0.62 (0.478∼0.75)
Inferonasal GCIPL	0.51 (0.35∼0.66)	RNFL2′	0.52 (0.378∼0.66)
Superonasal GCIPL	0.65 (0.50∼0.80)	RNFL1′	0.54 (0.39∼0.68)

*The area under the receiver operating characteristic curve (AUC) above 0.85.

*RNFL, retinal nerve fiber layer; GCIPL, ganglion cell plus inner plexiform layer.*

## Discussion

In this study, we retrospectively reviewed the longitudinal changes in the pRNFL and GCIPL among patients with adolescent myopia and juvenile glaucoma and compared the changes caused by progressed myopia and progressed glaucoma. Both progressed myopia and glaucoma could lead to a decrease in the average, superior, and inferior pRNFL. Temporal pRNFL showed an increase in the progressed myopia group, but decrease in the progressed glaucoma group. The GCIPL became thinner during follow-up in both the progressed myopia and glaucoma groups, and only the annual change rate of the inferotemporal and superotemporal GCIPL had a statistically significant difference between the two groups. The AUC analysis revealed that among the pRNFL and GCIPL parameters, the annual change rate of temporal pRNFL, inferotemporal GCIPL, and pRNFL at the 7, 8, 9, and 10 o’clock sectors had better diagnostic ability to differentiate progressed glaucoma from progressed myopia.

The previous cross-sectional studies (14–16) have confirmed that the distribution of pRNFL in eyes with myopia, especially in eyes with high myopia, is different from that in non-myopic eyes. Eyes with myopia displayed thicker temporal pRNFL and thinner superior, inferior, and nasal pRNFL (15, 16). Recently, Lee et al. (17) reported a decrease of the pRNFL on adult high myopia with time in a 2-year longitudinal study. However, the longitudinal databases of pRNFL in adolescents are still limited. This study revealed that with the progression of myopia in adolescents, the whole pRNFL decreased and shifted to the temporal quadrant gradually, which leads to an increase in temporal pRNFL and a reduction in the superior, inferior, and nasal pRNFL. There might be several factors which account for the result. First, the reduction in the whole pRNFL thickness may be due to the globe elongation that leads to mechanical stretching and thinning of the retina resulting from the progression of myopia over time (18). The previous studies have proved that the increase of myopia refraction is positively correlated with the increase of axial length (19, 20). In this study, though axial length data were lacked, the increase of myopia refraction might indirectly reflect the increase of axial length. Second, peripapillary atrophy, optic disk tilting, and rotation are very common in myopic eyes in Chinese population (21). As the progression of myopia, the occurrence of peripapillary atrophy, optic disk tilting, and rotation may lead to the redistribution of pRNFL (22) and a more temporally positioned superior or inferior peak locations (23). Third, the larger retinal vessels play a significant role in peripapillary RNFL thickness, since they comprise 13% of the total RNFL thickness (24). The previous study has reported that myopia status played effect on the superior and inferior vessel positions (25). The peaks of peripapillary RNFL profiles may change with superior and inferior vascular structures in myopia eyes (26).

A study conducted by Kanamori et al. (27) revealed that the RNFL thickness at the 7 o’clock position had the widest areas under the ROC curves for all parameters for early glaucomatous eyes (0.873). Lloyd et al. (28) found that the inferotemporal quadrant was the most common location for GP in ocular hypertension and early glaucoma. Kim et al. (29) reported that the progression of glaucoma had significantly higher rates on inferotemporal and supertemporal RNFL thicknesses. This study revealed in the glaucoma group, the greatest reduction in pRNFL was located at 7 o’clock sections These results confirm that the inferotemporal pRNFL is most vulnerable to glaucomatous damage. Moreover, despite the increase in refractive error in the glaucoma group being comparable to that in the myopia group in this study, the temporal pRNFL still became thinner in the glaucoma group. The greatest reduction region was more temporal in the glaucoma group than in the myopia group. The AUC analysis also showed that the annual pRNFL change rate at the 10, 8, 9, and 7 o’clock sections and also the temporal RNFL was above 0.90. The result may indicate that when considering the influence of myopia on the pRNFL, the change in temporal pRNFL may provide valuable information in addition to inferotemporal pRNFL in detecting glaucoma from myopia eyes.

A longitudinal study conducted by Lee et al. (30) reported that highly myopic eyes had thinner GCIPL and a significantly greater reduction in GCIPL over 3 years. Studies (31, 32) demonstrated that GCIPL thicknesses were significantly thinner with decreasing SE and also significantly thinner with increasing axial length. Miller et al. (13) found patient with high myopia experience thinning of non-temporal parameters ganglion cell-inner plexiform layer. Our study displayed similar results that both myopia and GP could lead to a decrease in the average and sectoral GCIPL, but the annual change rate of inferotemporal GCIPL in the GP group was much greater than that in the MP group. It has been proposed by Mill et al. that myopia-induced thinning of GCIPL is ubiquitous and most-pronounced with higher degrees of myopia, but that temporal GCIPL is least susceptible. A study (33) investigated the glaucoma detection ability of the macular GCIPL in myopic preperimetric glaucoma (PPG) and found that the best parameter for discrimination of myopic PPG from myopic healthy eyes was inferotemporal macular GCIPL thickness. Our study’s result was consistent with these results. The inferotemporal GCIPL corresponds to the inferotemporal and inferior pRNFL structures, which has been proven to be the region most vulnerable to glaucomatous damage. The reduction of inferotemporal GCIPL provided further evidence of the impairment of inferotemporal and inferior pRNFL. The AUC analysis reveals that the annual change rate of inferotemporal GCIPL had the greatest AUC among all sectoral GCIPL parameters in differentiating glaucoma from myopia confirmed inferotemporal GCIPL provide additional information beyond the temporal pRNFL in detecting GP.

There are some limitations in our study. Given that axial length measurement was not routinely performed for the patients involved in the study, we used SE. Despite associations that have been shown between SE and axial length (19, 20), and the pRNFL was also reported to be thinner with negative SE and longer AL (34), Guo et al. (35) reported significant axial elongation with minimal refraction changes among Chinese preschoolers, the effect of axial length change on pRNFL and GCIPL in adolescents still needs further study. Another limitation is that we did not collect data from a large sample size, which would have increased the power, and possibly strengthened the findings of our study. Despite this weakness, this study reported the preliminary results of the longitudinal changes in pRNFL and GCIPL between progressive myopia and glaucoma among adolescents and revealed that even though both GP and MP could cause reduction of the whole pRNFL and GCIPL in adolescents, the loss patterns of pRNFL and GCIPL were different between the two groups. The temporal (7, 8, 9, and 10 o’clock sectors) pRNFL plus the inferotemporal GCIPL can help to distinguish the loss of pRNFL and GCIPL caused by glaucoma or MP.

## Data Availability Statement

The raw data supporting the conclusions of this article will be made available by the authors, without undue reservation.

## Ethics Statement

The studies involving human participants were reviewed and approved by the Ethics Review Committee of the Zhongshan Ophthalmic Center. Written informed consent to participate in this study was provided by the participants’ legal guardian/next of kin.

## Author Contributions

HX: designing the study, analyzing the data, and writing the manuscript. YZ: collecting the data and editing the important part of the manuscript. YL and XX: collecting and analyzing the data. XL: designing the study and editing the important part of the manuscript. All authors met the ICMJE authorship criteria.

## Conflict of Interest

The authors declare that the research was conducted in the absence of any commercial or financial relationships that could be construed as a potential conflict of interest. The handling editor declared a shared affiliation with the authors at the time of the review.

## Publisher’s Note

All claims expressed in this article are solely those of the authors and do not necessarily represent those of their affiliated organizations, or those of the publisher, the editors and the reviewers. Any product that may be evaluated in this article, or claim that may be made by its manufacturer, is not guaranteed or endorsed by the publisher.
